# Assessing Sensitivity and Specificity of Surveillance Case Definitions for Zika Virus Disease

**DOI:** 10.3201/eid2304.161716

**Published:** 2017-04

**Authors:** Angela Chow, Hanley Ho, Mar-Kyaw Win, Yee-Sin Leo

**Affiliations:** Tan Tock Seng Hospital, Singapore

**Keywords:** Zika virus disease, Zika virus, viruses, case definitions, Centers for Disease Control and Prevention, World Health Organization, Pan American Health Organization, European Centre for Disease Control and Prevention, Singapore Ministry of Health, sensitivity, specificity, positive likelihood ratio, performance, surveillance, rash, Singapore

## Abstract

We evaluated performance of 5 case definitions for Zika virus disease surveillance in a human cohort during an outbreak in Singapore, August 26–September 5, 2016. Because laboratory tests are largely inaccessible, use of case definitions that include rash as a required clinical feature are useful in identifying this disease.

Zika virus infections in humans were first reported in Nigeria, Uganda, and Tanganyika (now Tanzania) in 1951–1952 (*1*,*2*). Until 2006, sporadic cases and small clusters of Zika virus infections were reported (*3*). In 2007, the first major outbreak occurred on Yap Island, where ≈1/5 infected persons were symptomatic, predominantly with rash, fever, arthralgia, and conjunctivitis (*4*). In a recent outbreak in Brazil in 2015, similar signs and symptoms predominated (*5*). Rash (67%), fever (64%), arthralgia (29%), myalgia (24%), headache (22%), and conjunctivitis (21%) were the 6 most common signs and symptoms reported during January 1964–February 2016 (*3*).

Unlike dengue virus (a related flavivirus), Zika virus was not considered to be a major pathogen until recent reports of its association with Guillain-Barré syndrome and microcephaly (*6*). Thus, there is little information on the performance of surveillance case definitions for detection of Zika virus disease.

Responding to the rapidly evolving Zika virus epidemic to guide surveillance for Zika virus disease, the US Centers for Disease Control and Prevention worked with the Council of State and Territorial Epidemiologists (CSTE) to approve an interim definition in February 2016 and a final case definition in June 2016 for noncongenital Zika virus disease as >1 of the following signs or symptoms: acute onset of fever, maculopapular rash, arthralgia, and conjunctivitis (*7*). The interim case definition (February 2016) of the World Health Organization (WHO) for suspected Zika virus disease includes rash or fever and >1 of the following signs or symptoms: arthralgia, arthritis, and conjunctivitis (nonpurulent/hyperemic) (*8*). The case definition of the European Centre for Disease Prevention and Control (ECDC) includes rash and optional symptoms in the WHO definition plus myalgia (*9*). The case definition of the Pan American Health Organization (PAHO) includes rash and >2 of the following signs or symptoms: fever, conjunctivitis (nonpurulent/hyperemic), arthralgia, myalgia, and periarticular edema (*10*).

The first outbreak of Zika virus disease in Singapore occurred in August 2016 (*11*). Singapore is a densely populated tropical country to which dengue fever is endemic. With the identification of the first local case of Zika virus disease, the Singapore Ministry of Health (MOH) initiated active case finding (*12*,*13*). The MOH recommended Zika virus screening for persons with fever and maculopapular rash, and 1 of the following: arthralgia, myalgia, headache, and nonpurulent conjunctivitis.

Clinical criteria for disease surveillance are a balancing act for satisfying 2 potentially conflicting needs: sensitivity and specificity. A more sensitive case definition will identify a larger proportion of true cases, but at the cost of finding a large number of cases from other causes. In comparison, a more specific case definition will provide a more accurate description of true cases, but at the expense of missing true cases (*14*).

## The Study

We evaluated the performance of surveillance case definitions for Zika virus disease recommended by the CSTE, WHO, PAHO, ECDC, and the Singapore MOH by using a cohort of 359 adult patients with suspected Zika virus disease who came to the Institute of Infectious Diseases and Epidemiology, Tan Tock Seng Hospital, Singapore, the national referral center for Zika virus disease during the containment phase of the Zika virus outbreak during August 26– September 5, 2016. All adults living or working in the outbreak area who were sick and had symptoms that partially or fully met the MOH definition were screened for Zika virus disease.

At their first visit to the hospital, all patients had their signs and symptoms documented, and blood and urine samples were obtained for detection of Zika virus nucleic acids by reverse transcription PCR (RT-PCR) (*15*). Parallel testing in the hospital laboratory and at the National Public Health Laboratory (Singapore) was conducted to maximize sensitivity and negative predictive values to rule out Zika virus infection.

A total of 42.0% of the cohort had Zika virus infection confirmed in blood (4%), urine (36%), or both (60%) samples (Table 1). Most (80%) infected and noninfected patients were tested <5 days after illness onset (infected patients, mean 3.6 days; noninfected patients, mean 4.6 days). Infected and noninfected patients were similar in age and sex. No female patients were pregnant. Among Zika virus–infected patients, rash (93.3%) was the most common symptom, followed by fever (79.2%) and myalgia (42.3%). Headache, arthralgia, and conjunctivitis were reported in <25% patients with Zika virus disease. Pruritus (11.4%) and gastrointestinal symptoms (6.7%) were relatively uncommon. For patients not infected with Zika virus, fever (86.2%) was the most common symptom, followed by myalgia (59.1%) and rash (44.8%).

**Table 1:** Clinical characteristics of an adult cohort with suspected Zika virus disease, Singapore, August 26–September 5, 2016*

Characteristic	Zika virus positive, n = 149	Zika virus negative, n = 210
Demographic data		
Mean age, y (SD)	38.1 (14.2)	34.2 (12.1)
Sex		
M	92 (61.7)	129 (61.4)
F	57 (38.3)	81 (38.6)
Ethnicity		
Chinese	109 (73.2)	122 (58.1)
Malay	15 (10.1)	17 (8.1)
Indian	9 (6.0)	24 (11.4)
Other	16 (10.7)	47 (22.4)
Singapore residents	113 (75.8)	131 (62.4)
Signs and symptoms at presentation		
Rash	139 (93.3)	94 (44.8)
Fever	118 (79.2)	181 (86.2)
Myalgia	63 (42.3)	124 (59.1)
Headache	35 (23.5)	75 (35.7)
Conjunctivitis	35 (23.5)	32 (15.2)
Arthralgia	34 (22.8)	50 (23.8)
Pruritis	17 (11.4)	17 (8.1)
Any gastrointestinal symptom†	10 (6.7)	25 (11.9)
Fulfilled case definition		
United States‡	149 (100.0)	206 (98.1)
World Health Organization§	57 (38.3)	64 (30.5)
PAHO¶	73 (49.0)	50 (23.8)
ECDC#	83 (55.7)	55 (26.2)
Singapore Ministry of Health**	81 (54.4)	51 (24.3)

*Values are no. (%) except as indicated. ECDC, European Centre for Disease Prevention and Control; PAHO, Pan American Health Organization. †Nausea, vomiting, diarrhea, or abdominal pain. ‡Definition used in the United States (interim, February 2016; confirmed, June 2016) states that clinical criteria for noncongenital Zika virus disease are defined as >1 of the following: acute onset of fever, maculopapular rash, arthralgia, and conjunctivitis. §Interim case definition (February 12, 2016) includes rash or fever and >1 of the following signs or symptoms: arthralgia or arthritis, or conjunctivitis (nonpurulent/hyperemic). ¶Interim case definition (April 1, 2016) includes rash with >2 of the following signs or symptoms: fever, conjunctivitis (nonpurulent/hyperemic), arthralgia, myalgia, or periarticular edema. #Interim case definition (March 17, 2016) includes rash with or without fever and >1 of the following signs or symptoms: arthralgia, myalgia, or conjunctivitis (nonpurulent/hyperemic). **Case definition (August 2016) includes fever and rash and >1 of the following symptoms: headache, myalgia, arthralgia, or nonpurulent conjunctivitis.

The case definition recommended by CSTE for use in the United States (US definition) had a sensitivity of 100% and a specificity of 2% in detecting Zika virus in the cohort (Table 2). The WHO case definition had the lowest sensitivity (38%). The Singapore MOH case definition had a sensitivity of 54% and a high specificity of 76%, and performed well in diagnosing Zika virus disease (positive likelihood ratio [LR+] 2.2, 95% CI 1.7–3.0). The performances of PAHO (LR+ 2.1, 95% CI 1.5–2.8) and ECDC (LR+ 2.1, 95 % CI 1.6–2.8) case definitions were similar.

**Table 2:** Performance of case definitions for diagnosing Zika virus infection in a human cohort during an outbreak, Singapore, August 26–September 5, 2016*

Case definition	Sensitivity, %	Specificity, %	PPV, %	NPV, %	LR+ (95% CI)	LR– (95% CI)
United States	100	2	42	100	1.02 (1.00–1.04)	0
WHO	38	70	47	61	1.3 (0.9–1.7)	0.9 (0.8–1.0)
PAHO	49	76	59	68	2.1 (1.5–2.8)	0.7 (0.6–0.8)
ECDC	56	74	60	70	2.1 (1.6–2.8)	0.6 (0.5–0.7)
Singapore MOH	54	76	61	70	2.2 (1.7–3.0)	0.6 (0.5–0.7)

*ECDC, European Centre for Disease Prevention and Control; LR, likelihood ratio; MOH, Ministry of Health; NPV, negative predictive value; PAHO, Pan American Health Organization; PPV, positive predictive value; WHO, World Health Organization; +, positive; –, negative.

## Conclusions

Despite increasing incidence of Zika virus disease and its spread across the Americas and Asia, there is no internationally adopted common clinical criteria for the surveillance of this disease. We report a large outbreak cohort of patients with suspected Zika virus infection and comprehensive documentation of clinical symptoms and parallel RT-PCR conducted on blood and urine samples for these patients by 2 laboratories. Evaluation of the performance of surveillance case definitions in such a cohort would provide useful findings that would contribute to development of guidance for Zika virus disease surveillance.

Diagnosis of Zika virus disease remains suboptimal because of limited availability of confirmatory testing by RT-PCR during acute illness and cross-reactivity of serologic tests for Zika virus with other co-circulating flaviviruses (*3*,*4*). Thus, a good discriminatory clinical criteria for disease surveillance is crucial for prevention and control of Zika virus transmission.

The US case definition would identify all Zika virus infections and be useful for prevention of autochthonous transmission by imported cases. However, because this definition is not specific, considerable resources would be required for confirmatory testing of identified cases. The definition requires laboratory testing to report a case. Thus, sensitivity of the definition is most likely appropriate in the US setting. Conversely, the WHO case definition might miss 60% of Zika virus infections. For Zika virus disease surveillance in the absence of commercially available diagnostic laboratory tests, case definitions incorporating rash as a required clinical criteria, such as the PAHO, ECDC, and Singapore MOH case definitions, would be useful (LR+ >2), although ≈50% (range 44%–51%) of cases of Zika virus disease could be missed.

The main limitation of this study is that it included only adults. However, the small number of children infected with Zika virus during the containment phase of the outbreak in Singapore had symptoms similar to those for adults (A. Chow et al., unpub. data). Some Zika virus infections could have been misclassified as noninfections because RT-PCR could have missed infections late in the illness course or after development of antibodies against Zika virus.

In conclusion, we evaluated the performance of 5 case definitions for Zika virus disease surveillance. In the current effort to halt transmission of this virus worldwide, and with laboratory tests being largely inaccessible, use of surveillance case definitions that include rash as a required clinical criteria would provide a high yield in identifying Zika virus disease.
